# Ghosts in the labour market: perceived health implications of informal labour in Australia

**DOI:** 10.1093/heapro/daae089

**Published:** 2024-08-02

**Authors:** Miriam van den Berg, Fran Baum

**Affiliations:** Stretton Health Equity, Stretton Institute, University of Adelaide, North Tce, Adelaide, South Australia 5005, Australia; Stretton Health Equity, Stretton Institute, University of Adelaide, North Tce, Adelaide, South Australia 5005, Australia

**Keywords:** informal employment, social determinants of health, employment conditions, commercial determinants of health, informal work, neoliberalism, high-income countries

## Abstract

Employment conditions are important social and commercial determinants of health. Informal employment—also known as ‘cash-in-hand’ and ‘undeclared’ work—is a discrete employment condition that has salience around the world. Fuelled by neoliberal ideology, informal employment has become increasingly common in high-income countries. Public health research concerning the health of informal workers comes largely from low- and middle-income countries, where the phenomenon is more visible. There has been little research on the health effects of informal employment in high-income countries including Australia. Twenty-nine workers aged 18 years and older, who were undertaking informal work activities, were recruited using social media and an online marketplace in Tarndanya (Adelaide-Kaurna Country), Australia. Qualitative narrative data, demographic profiles, and physical and mental health scores were collected. Most informal workers reported unfair and indecent employment conditions including job insecurity, low income, coercion, and lack of respect and dignity at work, and were often exposed to unsafe and unhealthy work environments. Workplace injuries and exposure to occupational hazards were common; and Physical and Mental Component Scores were poorer among informal workers when compared to the population of South Australia as a whole. With informal employment in Australia described as part of a ‘significant, pervasive, damaging and growing’ problem, there is a need for a health promotion lens over industrial relations policies in the interest of creating equitable access to fair and decent work.

**Contribution to Health Promotion**

- Employment and work conditions are important social and commercial determinants of health and recognized as a source of health for people by the Ottawa Charter for Health Promotion.
- This study provides insight into one particular type of employment condition known as informal employment, which has significant consequences for health.
- This study makes an important contribution to an understanding of informal employment and health in a high-income country—a little studied area.
- The article highlights the need for health promoters to play a role in advancing the fair and decent work agenda.

## BACKGROUND

The Ottawa Charter for Health Promotion recognizes that ‘work should be a source of health for people’ and that ‘the way society organises work should help create a health society’ (WHO, 1986). Employment conditions are important social and commercial determinants of physical and mental health, because they define the rights-related details of the contractual arrangements (e.g. wages, working hours, length of tenure, worker rights) between employers and employees. Informal employment is one type of employment condition on the decent through unacceptable work spectrum (McCann and Fudge, 2017)—others include full employment, precarious employment, unemployment, child labour, bonded labour and slavery (Benach *et al.*, 2010a). Informal employment is the most common form of employment in the world [International Labour Organization (ILO), 2018], with the agricultural, domestic and construction industries most likely to hire informal labour (OECD, 2023). As such it is important for health promotion to understand its health impact.

Informal employment is a complex phenomenon which, from a definitional perspective, crosses boundaries with precarious employment and may involve workers who also work in the formal sector (Kreshpaj *et al*., 2020; Cui *et al*., 2022). The unique features of informal employment are tied to the concept of invisibility, in that informal workers are involved in unrecognized or unregulated employment relationships, irrespective of whether they work in economic units in the formal or informal economy (Benavides *et al*., 2022; Cui *et al*., 2022). Informal work includes legal production activities that are deliberately concealed from public authorities for reasons relating to taxation avoidance; avoiding having to meet certain legal standards such as maximum hours, safety or health standards; and evading compliance with certain administrative procedures, such as completing statistical questionnaires or other administrative forms (OECD, IMF, ILO and CIS STAT, 2002).

The ILO estimates that two billion people (61% of the world’s employed population) make their living in the informal economy, undertaking a wide range of occupations (ILO, 2018). Although more common and visible in low- and middle-income countries (LMICs), informal labour has global prominence. In low-income countries, informal employment is reported to account for 89% of total employment, while in high-income countries, it constitutes around 16% of all employment (OECD, 2023). With the expansion of insecure employment in high-income countries (Mai *et al*., 2023), it would seem probable that informal employment will continue to grow.

Informal employment denies workers ‘fundamental principles and rights at work, that put at risk the lives, health, freedom, human dignity and security of workers or keep households in conditions of poverty’ (ILO, 2015). Reflecting the size of the informal economy, studies reporting on the health consequences of informal employment have predominantly been carried out in LMICs (van den Berg, 2020; Benavides *et al*., 2022). The findings of these studies have informed the development of various models and frameworks for explaining the relationship between employment and health. For example, Benach *et al*. (Benach *et al.*, 2010b) and Muntaner *et al*. (Muntaner *et al*., 2010) outline the macro-level (e.g. power relations and policies) and micro-level (e.g. working conditions: work quality, hazards) pathways linking different employment conditions (including informal employment) and health. Benavides *et al*. (Benavides *et al*., 2022, p. 170) propose a conceptual framework which unifies employment (e.g. salary, social rights, working hours) and working conditions (e.g. physical and chemical exposures, high demand, low control), noting that informal employment ‘generally represents the worse scenario’ for workers because informal workers experience both poor employment as well as poor working conditions.

Studies and reviews of health and informal employment in high-income countries are limited (Walter *et al*., 2002; Gutberlet *et al*., 2009; Ahonen *et al*., 2010; Salgado *et al*., 2012; Panikkar *et al*., 2015; Julià *et al*., 2016; Julià *et al*., 2019; Aronsson *et al*., 2023) and report mixed results (Lee and Di Ruggiero, 2022). For example, a study of day labourers in the USA found that mental and physical health scores were lower for this sample compared to normative data (Salgado *et al*., 2012). Julià *et al*. (Julià *et al*., 2019) reported that informal workers in the EU had poorer psychological well-being but better self-rated health and fewer health problems in the past 12 months compared to formal workers; while Montero-Moraga *et al*. (Montero-Moraga *et al*., 2020) found that women in informal employment in Spain reported poorer self-perceived health status than those under other employment profiles. Other studies report on the high rates of exposures to hazardous working conditions (Walter *et al*., 2002; Gutberlet *et al*., 2009; Panikkar *et al*., 2015), including psychosocial factors (Ahonen *et al*., 2010).

Researchers undertaking high-income country studies concur that power vested through social hierarchies based on gender, age, race and social class, and sociopolitical context have important ramifications for explaining the relationship between informal employment and health (Montero-Moraga *et al*., 2020; Rodriguez-Loureiro *et al*., 2020; Lee and Di Ruggiero, 2022; Aronsson *et al*., 2023). The size of informal economies around the world has been linked to the type of welfare regimes adopted by countries (Chung *et al*., 2010; Rodriguez-Loureiro *et al*., 2020). Less equal societies and countries with lower levels of welfare protection have been found to be strongly correlated with the extent and nature of the informal sector (Williams, 2015).

This article aims to contribute to the limited literature on informal employment and health in high-income countries by presenting the findings of a study conducted in Australia that investigated the following research question: What do informal workers in Australia perceive are the health effects of their involvement in informal employment? The study provides insight into participants’ experiences in the informal employment sector and highlights the need for health promoters to advocate for employment environments that are conducive to health and well-being.

## METHODS

This study was approved by the Flinders University Social and Behavioural Research Ethics Committee (Application # 6786) before data collection and analysis.

Data collection was carried out in 2018–2020 in the northern suburbs of Tarndanya (Adelaide-Kaurna Country), Australia. As the study cohort was considered ‘hard to reach’ (Shaghaghi *et al*., 2011), participant recruitment involved a combination of passive and active recruitment, purposive and snowball sampling (Valerio *et al*., 2016), and the use of social media (Arigo *et al*., 2018). Informal workers, receiving cash-in-hand for undertaking legal production activities, aged 18 years and over and who had worked in this capacity for at least 6 months cumulatively or actively for the past 6 weeks, were invited to participate in the study. People in other forms of precarious work that operated within recognized legal/policy frameworks were excluded. All participants completed a consent form prior to participation.

Josselson’s (Josselson, 2013) approach to narrative inquiry was used to interview study participants. Narrative inquiry is concerned not only with individuals’ experiences in the world but also the social, cultural and institutional narratives which interact and shape these experiences (Clandinin, 2013). In this way, the approach aligns with the framing of health as a socially determined concept, in which employment has a key role. Interview questions centred on entry into and navigation of the informal employment market, experiences, benefits and challenges of this form of employment, and perceived health and well-being impacts. Interviews took up to 120 minutes, were voice recorded and transcribed verbatim. All participants were given a $50 voucher to compensate for their time.

Participants were also asked to answer demographic and SF-12 questions—a validated and widely used Short-Form Health Survey derived from the SF-36 to asses self-rated physical and mental functioning, pain and impairment, which make up MCS (Mental Component Summary) and PCS (Physical Component Summary) scales (Ware *et al*., 1996). The US version of the SF-12 is considered appropriate for use among the Australian population (Wilson *et al*., 2002).

The data were deidentified and pseudonyms were assigned to maintain participant confidentiality during analysis. Narrative analysis of interview data was conducted in two ways: holistically, where stories were considered as a whole and sections of text were interpreted with respect to the other parts; and categorically, where abstract sections of a story were compared with other stories (Ollerenshaw and Creswell, 2002; Josselson, 2011). Holistic narratives for each participant were reconstructed as an output of the study. Narrative data were further analysed using a deductive yet flexible coding process that drew on existing theoretical frameworks of informal employment and health (Benach *et al*., 2006). NVivo qualitative data analysis software (QSR International) aided in this analysis.

Quantitative data were managed and analysed using Microsoft Excel. Individual SF-12 MCS and PCS scores were calculated using the process outlined by Ware *et al*. (Ware *et al*., 1996).

Strategies to promote study quality throughout data collection and analysis involved appropriate sampling methods, lengthy data collection processes, data saturation, member checking, thick and rich descriptions, peer de-briefing, an audit trail, and researcher reflexivity and critical reflection.

Twenty-nine people participated in the study. Their demographic profile is provided in Table 1.

**Table 1:** Demographic profile of study participants

Demographic variable	Subcategory	*n*
Gender	Female	13
	Male	15
	Transgender	1
Age (years)	18–20	4
	21–30	8
	31–40	7
	41–50	5
	51–60	4
	61–70	1
Country/region of birth	Australia	12
	Asia	7
	USA	1
	Europe	4
	Middle East	5
Residency status	Australian citizen	24
	Visa supported resident	5
Area of residence as indicated by Index of Relative Socio-economic Advantage and Disadvantage (IRSAD)^a^ decile rank within Australia	IRSAD = 1 (most disadvantaged)	12
	IRSAD = 3	10
	IRSAD = 7	2
	IRSAD = 9	3
	IRSAD = 10 (least disadvantaged)	2
Aboriginal or Torres Strait Islander	Yes	1
	No	28
Caring for a friend/family member	Yes	7
	No	22
Highest educational qualification	Left school at 15 years or less	2
	Left school after 15 years	4
	Left school after 15 years but still studying	3
	Certificate/Diploma	8
	Bachelor’s degree or higher	12
Personal annual income	Up to $12 000	9
	$12 001–$20 000	11
	$20 001–$40 000	8
	Don’t know	1
Personal sources of income	Informal employment only	4
	Informal employment and welfare support	16
	Informal and formal employment	3
	Informal and formal employment and welfare support	4
	Informal employment and study scholarship	2
Type of welfare support payments	Disability Support Pension	5
	Unemployment (Newstart)	9
	Parenting payment	1
	Youth allowance	1
	Study payment	1
	Carer payment	3
	None	9
Persons in household (18 years and older)	1	6
	2	14
	3	4
	4	3
	5	1
	6	1
Household annual income	Up to $12 000	3
	$12 001–$20 000	5
	$20 001–$40 000	6
	$40 001–$60 000	4
	$60 001–$80 000	3
	More than $80 000	2
	Don’t know	6
Perception about ability to manage financially	Living very comfortably	0
	Living quite comfortably	7
	Getting by	9
	Finding it quite difficult	11
	Finding it very difficult	2

^a^The IRSAD is a component of the Socio-Economic Indexes for Areas (SEIFA), which is the Australian Bureau of Statistics (ABS) ranking system for relative socio-economic advantage and disadvantage.

## RESULTS

Informal employment takes place as part of a broader set of socioeconomic and cultural circumstances, which are often also precarious in nature. We therefore begin this section by positioning the study findings in the context of participants’ life circumstances. Following this, we report on the nature of informal employment and workplace conditions, the perceived health effects of informal employment and participants’ health status.

### Precarious lives

Informal workers reported being ‘pushed’ and ‘pulled’ into informal employment opportunities by a combination of economic, social and cultural factors that combined with precarity in other areas of their lives. Some participants described being introduced to informal work from a young age. For example, Mal (51) was working as a furniture removalist and said:

I did cash-in-hand work since I was a teenager. I worked for my parents in amusements, they did merry-go-rounds and stuff and they paid me cash-in-hand. [I’ve] done plenty of jobs where I got paid cash-in-hand.

Exclusion from the formal labour market and the need for income for daily life were the primary reasons why study participants were involved in informal work. Some participants reported being excluded from the formal labour market on the basis of their age (e.g. younger participants who lacked experience and older participants who believed they were ‘too old’), while others described prior life circumstances that lead them to pursue employment in the informal sector. For example, Brad (40) worked as a labourer (paving, brick laying, general construction) and avoided the formal sector because he had a criminal record that he did not want to disclose. He said:

…employers don’t like people that have been incarcerated…that’s why I tend to [get] cash-in-hand work.

Sue (55) worked as a seamstress, and explained how, through social connections, she had started her informal enterprise as a means to acquire additional income:

[after I left my husband and moved to this suburb] I was totally on my own and I had nothing when I moved in here, I had absolutely nothing. I didn’t have a stick of furniture to my name. I had to get a [no interest] loan from…which comes out of my fortnightly payments and leaves me very little for groceries and fuel and bits and pieces. I started going to [a non-government organisation] for lunch, and it was there that people were saying…can I do a bit of sewing and stuff.

More than two-thirds of study participants were receiving government welfare payments (for family caring responsibilities, disability, study or unemployment status) and had turned to informal employment due to the inadequacy of these payments. For example, James (39) worked as a DJ in pubs and clubs, and said:

I mean, the money’s not great, but, once again, it’s on top of [welfare payments] ...and [the welfare system] pays bugger all nowadays, so it’s, like, anything extra is a bonus, I suppose…

Participants also reported other life circumstances, for example associated with being a single parent, poor health, addictions, migration and visa status, that created pressures to acquire additional income. A number of female participants said they were involved in the informal sector because it enabled greater flexibility for combining work with family caring and other responsibilities. For example, Eliza (31) was a single mother and ran an informal childcare business from her home, which allowed her to generate an income, while at the same time caring for her own child. Five participants were living in Australia under temporary migration visas, which placed employment, welfare and health care access restrictions on their lives. Abdul (32), an Iranian-born asylum seeker and qualified accountant, struggled to gain recognition for his overseas-acquired qualifications in the formal labour market. Abdul had worked informally in Australia in agriculture, construction, food service and unloading containers.

Our narrative data revealed the interplay of informal employment with other social determinants of health, including poverty, insecure housing and homelessness, food insecurity, trauma in early childhood and young adulthood, low education, family breakdown and incarceration. Not only did workers identify direct and tangible effects of social factors on health (e.g. how insecure housing generates psychological distress), but they also spoke about the cumulative effect of multiple, long-term social stressors. Sam (43), for example, worked informally as a labourer, was a single father and was homeless at the time of the study, living in his car. He described difficult relations with his ‘wealthy’ family and reported that he feels downhearted and blue ‘most of the time’.

### Employment and workplace conditions

Study participants were employed in a wide range of occupations and industries including construction, agriculture, manual labouring, the arts, hospitality, mechanical work, domestic work, childcare, retail, recycling, delivery, administration, sewing, tutoring, and health and beauty (e.g. fitness, body piercing, tattooing). Informal work was carried out in the following locations: workers’ homes, public places, client’s homes, private business locations, sporting venues and formal workplaces. Few study participants demonstrated sound knowledge of occupational health and safety (OHS) standards, with agency for personal protective equipment resting with individual workers.

Informal employment arrangements were characterized by casual agreements (generally verbal) between employees and employers, or self-employment (sometimes also referred to as ‘own account workers’). Informal employment was acquired through social networks, online marketplaces, advertisements in public places and through proactive approaches. Study participants did not partake in formal recruitment processes, training nor receive relevant documentation (e.g. a position description, employment contract or payment advice). Most study participants expressed a desire for alternative work arrangements, with greater security and certainty—while also regarding this as an unachievable aspiration. For example, Trevor (41), who worked in construction, said he could not imagine having a ‘decent’ job:

[I’d] love to worry about nothing. Go in and go to work, knock off…[I’d] be dreaming to get somewhere with good conditions.

Participants working for an employer were often unclear about the days and hours of work expected of them, saying that it varied considerably and was poorly organized. Income ranged from AUD$7.00 per hour for food services work to AUD$50.00 per hour for construction. Few informal workers were aware of legislated award rates and none reported being union members. Participants did not have access to entitlements such as superannuation, personal/sick/carers leave and workers compensation insurance.

Self-employed participants commonly presented during interviews as lacking in confidence, underselling their occupational experience and capabilities, and some stated that they settled for lower levels of pay then they admitted being satisfied with. As study participants had no job security, it was common, particularly for those who had been working informally for a long time, to have had a wide variety of different occupations and to consider themselves ‘a jack of all trades’ with a high level of job mobility.

There were several examples of exploitation and discrimination reported by study participants. Hospitality worker, Diego (34), said that employers can ‘treat you as slaves’, demanding long hours and intense conditions in busy restaurant kitchens. He was born in South America and arrived in Australia 3 years ago. He said,

…me, as a foreigner, we don’t know that stuff [pay and conditions]. If he [the boss] tells you when you arrive, ‘This is a minimal wage’, or ‘This is the proper way’ [you believe him] …you are forced to do whatever…to survive, pay the bills and all that.

Similarly, food industry worker, Sophie (18), said she asked her employer to formalize her employment arrangement but that her request was ignored. Sophie was a fulltime university student of Korean heritage. She worked 25 hours per week in a takeaway food business. Sophie said she had to monitor her pay closely to ensure she received what she believed she was entitled to—a process she found both unfair and distressing. Sophie said:

…they [business owners] get away with it ‘cause some people who work there can’t speak English so it’s like you can work here or just not work at all, never get a job. So, they can get away with like low pay…rates.

While most participants described feeling trapped in the informal sector and having little choice in their working lives, some participants focused on the benefits of informal employment including the additional income, enhanced self-determination and flexibility. For example, Sally (48) had extensive experience in a wide range of occupations in the informal sector. She reported being diagnosed with several physical health problems, and said that informal work allowed her to determine when she felt able to work and when her ill-health prevented it:

…what control means for me is…nominating for myself what days I work, what days I rest…when you are working to someone else’s schedule…there are expectations additional to what’s needed to do the job, about what a good worker looks like. And I don’t always fit those without a lot of extra effort. So, this is a way of meeting my needs.

For three males aged under 30 years, informal employment conditions were considered to provide more benefits than disadvantages. These informal workers described the positive psychological sensations associated with perceptions of control over their working lives, taxation avoidance, being paid ‘in cash’ and undertaking physically demanding work. Ali, 28, who worked in the entertainment industry, as well as hospitality and ‘laborious heavy-duty type jobs…tiling, concreting, different trade jobs’, said:

…that feeling of getting paid on the spot and then seeing the money as opposed to its just electronically there…it makes me feel pretty good because it’s all hard, like boys physical work. So, at the end of the day when you get paid, that’s because you seeing the money like after you’ve finished …[it’s] a good feeling that you earn it… [there are] more benefits…than non-benefits.

### Workplace hazards and self-reported health effects of informal employment

The physical, chemical, ergonomic and psychosocial hazards participants reported being exposed to whilst undertaking informal work are outlined in Table 2, along with the health conditions they experienced. Exposure to chemicals and other hazardous substances, loud noise and other physical hazards, heavy lifting, lack of work breaks, long work hours, prolonged standing/walking/running or other static positions, and undertaking work without adequate training were all perceived to affect informal workers’ physical health.

**Table 2:** Exposures and health conditions reported by informal workers

Exposures during informal employment as reported by participants	
Chemical	Cleaning chemicals, horticultural chemicals
Ergonomic	Long hours without breaks, prolonged standing/walking/running or static positions, heavy lifting and manual labouring
Physical	Asbestos dust, clay dust, sun and heat exposure, loud noise, sharp objects, construction materials, heavy objects, heights, workplace equipment (e.g. sewing machine, hot appliances), violence and physical encounters, contaminated waste, biological matter
Psychosocial	Invisibility, job insecurity, not paying tax, low pay and lack of entitlements, unfair power relations (lack of respect, exploitation), irregular work hours, misrepresenting work activities, violence, lack of work breaks

Health conditions during informal employment as reported by participants	
Mental health and well-being	Anger issues, anxiety, depression, isolation, mental distress, night terrors
Physical	Back, shoulder, neck and knee pain (in some cases, arthritis) and other bodily pain, breathlessness (associated with anxiety), concussion, electric shocks to the body, enuresis/encopresis, eye and skin irritation, fatigue, foot pain and plantar fasciitis, hearing loss, headache (sometimes associated with mental distress), inhalation of hazardous substances (e.g. asbestos, clay dust), insomnia (sometimes associated with mental distress), joint dislocation, puncture wounds, cuts and burns, sprains, torn muscles, sciatica and bruising

Mental health was impacted by aggressive behaviour in the workplace (being yelled at), employment invisibility (including feelings of deception in relation to tax evasion) and insecurity, lack of work breaks, unfair pay, unsuitable work (i.e. lack of skills/physical ability to undertake tasks), and unreasonable responsibility and expectations. For example, Li (26) was from Indonesia and studying in Australia. She worked in a supermarket during evenings, where she was given sole responsibility for running the business. Li reported feeling unsafe at night but said that raising her concerns would cost her the position. She said:

…especially Friday night [when] there are a lot of people who [have] been drinking and people who want to buy…cigarettes and yeah, there are scary looking people at night…if something happens you don’t have anywhere to go to, there’s only you.

Jamila (29) worked as a cleaner and demonstrated significant psychological distress associated with feeling like a lower-class citizen because of her role (‘dirty work’), the informal nature of the work and her gender:

…they [clients] just treat me like I’m someone really from a low class…they try to show me that I’m really the low-class person…it really hurts me, I really feel so much down. From my inside I feel so bad, I feel so down…when you do cash-in-hand they think you really need money and you’re not a good person you know…you’re cheating the government.

Study participants reported a wide range of physical and mental health conditions, which they associated with informal employment (Table 2). Agricultural workers reported suffering fatigue and headaches from long hours spent working at high temperatures both outdoors and in hothouses. Workers reported feeling pressured to work faster with few breaks and limited opportunities for rehydration. Hospitality workers reported similar psychosocial hazards. For example, Daisy (27) reported working 11-hour days for AUD$10.90 per hour (less than half the minimum wage), and suffered psychosocial distress and incontinence, when she was unable to take work breaks to go to the toilet:

…sometimes, I couldn’t go to toilet. Sometime I even pee myself or poo in myself because only me at a job 7:00am until 6:00pm and I don’t have lunchtime or breakfast time so I just eat when the customer left...I just brought a sign [that] said ‘I’m come back in five minutes’ and then I have to go and just feel awkward when you come out and see customer waiting there and you go to toilet…I feel it’s too much.

Numerous participants perceived that the stress associated with informal employment—including job insecurity, the impact of unequal power relations and avoiding the taxation system—contributed to a downward spiral in mental health and well-being. There were however three male participants (aged in their 20s) who demonstrated entrepreneurial motivations (in contrast, to the majority who worked informally as a means to meet the needs of daily life) and reported positive outcomes for their psychological well-being. Other participants spoke about the relative positive impact on mental well-being and the sense of purpose that informal work provided, and that it was an important alternative to unemployment or underemployment.

Some participants linked their employment to physical health benefits. For example, Moira (67) was an informal recycler, who rated her health as ‘excellent’. She said:

I’ve got the blood pressure of a…23-year-old. She said…health wise, I know for sure that if I wasn’t doing it, I wouldn’t be the person I am today.

These health benefits were attributed in part to physical nature of her occupation, which required many hours of walking.

### Self-reported health status and health behaviours

**Fig. 1:**
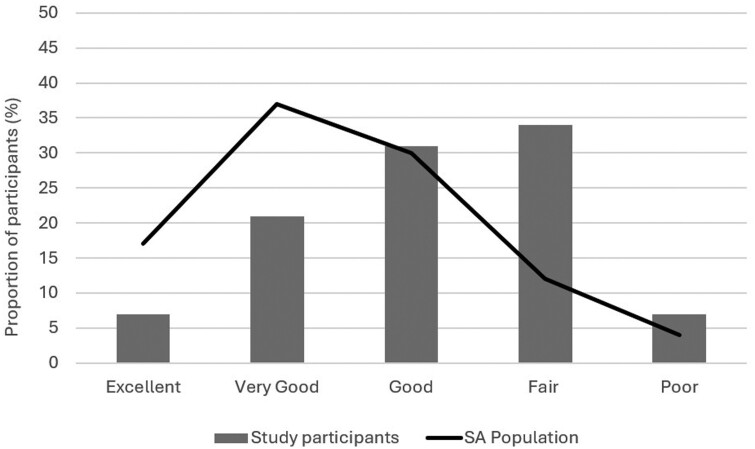
Frequency distribution for self-reported health rating (SF-12) by informal workers, 2018 (*n* = 29) and South Australian population, 2017 (*n* = 2977).

More than half of the study participants (58.6%) said they had good, very good or excellent health, while 12 participants (41.4%) rated their health as fair or poor (Figure 1). Although the study sample was not designed to be statistically representative of the wider population, South Australian population prevalences for health outcomes are provided to highlight the South Australian context. Self-rated health was poorer when compared to the population of South Australia as a whole (SAHMRI, 2018). Just over a quarter of participants (27.6%, *n* = 8) rated their health as very good or excellent in contrast to 54% of a sample of the general South Australian population (*n* = 2977); and 41.4% (*n* = 12) of participants rated their health as fair or poor compared to 16% of the South Australian population overall (SAHMRI, 2018).

There was wide variation about the mean for both PCS and Mental Component Summary (MCS) scores. Female participants reported a mean PCS sore of 41.99 (SD = 10.66) and males, 47.27 (SD = 7.30). The mean MCS score for females was 36.43 (SD = 14.75) and for males, 40.89 (SD = 12.06). Across all participants, some recorded very low health scores, particular MCS—the lowest for females was 13.6 and for males, 18.29. One participant identified as transgender and had a PCS of 58.34 and MCS of 20.52. Another participant had a very low PCS of 18.99; the participant had multiple chronic health problems.

A small number of participants reported smoking, alcohol and other drug use, and other health harming behaviours. Difficulties gaining access to health care was exacerbated by informal work arrangements, for example, participants reported challenges of acquiring sick leave certificates, taking time off for ill health and covering health care costs.

## DISCUSSION

This study adds to the literature on informal employment in high-income countries. It illustrates that informal employment does not provide fair and decent conditions conducive to good health and well-being. McCann and Fudge (McCann and Fudge, 2017) propose a multidimensional model of unacceptable forms of work, and this study found evidence that informal employment in Australia may be characterized by 10 out of 12 of the dimensions. These related to health and safety risks; inadequate and insecure income; employment insecurity; unpredictable and unprotected working times; lack of representation and voice; inadequate social protection; lack of human rights and dignity (with respect to gender, race and age); exclusion from legal protection; lack of leave entitlements; and lack of control over work organization.

The conditions of Australia’s welfare state may buffer some of the negative health and well-being effects of informal employment for eligible citizens. For example, informal work was rarely the sole source of income for study participants (a large proportion of participants received welfare support payments), many participants had access to some form of housing-related support (e.g. in the form of rent assistance) and most participants had access to Australia’s universal health care system. However, given that most study participants turned to the informal sector for reasons related to ‘survival’ (Lee *et al*., 2020), it is clear that the welfare state does not adequately provide for all its residents—and further, that it discriminates against particular groups. For example, while Australia rigorously regulates migration status, it inadequately oversees labour standards for migrant workers (Boucher, 2018). Five participants in this study were on temporary Australian visas and were ineligible for welfare assistance and access to Australia’s publicly funded health care system. Australia’s Fair Work Act 2009 sets out minimum terms and conditions for employment regardless of visa status; however, as informal employment is often invisible, there are no effective mechanisms by which such standards can be rigorously monitored and upheld. Clibborn (Clibborn, 2015) highlights how the intersection of Australian immigrant and employment policy has aided the growth of the informal migrant labour market, and the subsequent exploitation of these workers.

Informal labour is not a new phenomenon in Australia [Commonwealth of Australia (CoA), 2017] but its expansion has been promulgated by neoliberal ideology (Williams, 2017). Not dissimilar to other advanced capitalist countries, privatization, declining unionization and public sector cutbacks have weakened the position of labour (Stanford, 2019; Bessant and Watts, 2021; McGregor, 2022). The simultaneous growth of precarious employment and the weakening of industrial relations mechanisms have made it easier for ‘non-standard’ employment conditions to become the new norm (Campbell and Brosnan, 1999; Holley and Rainnie, 2012). Such neoliberal conditions naturally fuel the way for informal endeavours, as protective structures are dismantled, the marketplace takes prominence and productivity is prioritized over workers’ rights (Lee *et al*., 2020). The liberalization of labour possibly explains why a small number of study participants found informal employment rewarding, at least in the short term. Three young male study participants demonstrated what Temkin (Temkin, 2009) described as entrepreneurial characteristics: independent, optimistic and competitive. However, for the majority, informal employment was rarely the preferred choice. Most study participants expressed a desire for visible, secure and supportive employment conditions. Indeed, even for the entrepreneurial, upward mobility is a rare occurrence in the informal economy (Breman, 2013).

Irrespective of whether study participants reported benefits associated with their employment situation or not, none of the narrative accounts demonstrated acceptable working conditions (McCann and Fudge, 2017). Informal workers in this study reported being exposed to physical, chemical, ergonomic and psychosocial health risks. Although some of these exposures may be associated with occupation type and thus not exclusive to informal employment conditions, the lack of OHS measures and labour protection policies is likely to place informal workers at greater risk than those who are formally employed in the same occupations (Walter *et al*., 2002). When hazardous working conditions are combined with poor employment conditions, opportunities for good health through the mechanisms of decent work deteriorate (Benavides *et al*., 2022). A reflection of this is the self-rated health among study participants, which were similar to other South Australians living on very low incomes (below AUD$20 000) (SAHMRI, 2018).

To understand the relationship further however, it is important to extend the analysis of the consequences of informal employment and work on health and well-being beyond employment and working conditions. Benach *et al.* (Benach *et al.*, 2014) note the role of social precariousness in explaining the relationship between employment and working conditions and health. Narrative accounts offered by study participants outlined the status of other social determinants of health in their lives, including insecure housing and homelessness, trauma in early childhood and young adulthood, low education, family breakdown, social exclusion and incarceration [Commission on the Social Determinants of Health (CSDH), 2008]. Participants identified the direct effects of these social factors on health (e.g. how insecure housing generates psychological distress) as well as the cumulative effect of multiple, long-term social stressors (Emeny *et al*., 2021). All informal workers in this study experienced precarity in relation to other aspects of their lives, in addition to precarity in the field of employment. Thus, entry into, and the experiences and effects of informal employment need to be understood within the wider context of people’s lives as the interactive effects are likely to exacerbate negative health impacts.

Underhill and Rimmer (Underhill and Rimmer, 2015) explore the concept of layered vulnerability with respect to migrant workers in the Australian horticulture sector. In a similar way, some groups of informal workers in this study were more vulnerable than others on the basis of their social position, personal resources and powerful social structures. For example, informal workers in this study were more vulnerable if they had low levels of education and/or lacked citizenship status (i.e. a lower social position); did not have access to transport, housing and/or social networks (or other capital resources); and experienced particularly poor working conditions, discrimination and/or were subjected to onerous welfare system requirements (i.e. social institutions that are detrimental to health). These interactions make it challenging to isolate the impact of informal employment on health and well-being, and call for a multisector policy response.

That informal employment is associated with invisibility makes it additionally challenging for health-promoting policy responses to capture this working cohort. Books (2007, p. xvi) writes that when individuals are socially invisible, they ‘are devalued such that alleviating the difficult conditions of their lives is not a social priority’. In studying ‘invisible workers’ such as cleaners who do ‘dirty work’, Rabelo and Mahalingam (Rabelo and Mahalingam, 2018) noted that when people feel like they belong and are valued, they experience greater self-esteem, lower depression and anxiety. The invisible nature of informal employment—in combination with other sources of stress—may help explain some of the particularly low Mental Component Scores reported by participants. Not only are informal workers invisible by virtue of undocumented work they are undertaking but they also need to conceal their activity to avoid legal penalties. This type of ‘strategic invisibility’ required informal workers to make decisions about where and when to work to ensure anonymity and not attract interest from taxation, welfare and/or migration systems (Ham and Gerard, 2013). The prospect of ‘getting caught’ was a constant source of stress for informal workers.

## CONCLUSION

Employment and working conditions are an important source of health for a large proportion of the world’s population, and the way society organizes work are crucial social and commercial determinants of health that can have significant implications for population health and health equity (WHO, 1986). Health promoters can play an important role in advancing the fair and decent work agenda, not only through programs that help to create supportive work environments for health, but also by advocating for industrial relations policies and laws that create fair employment and working conditions, calling for greater accountability to uphold safe and healthy employment and working standards, and encouraging trade union membership. Importantly, such efforts must capture both those working informally in employee–employer agreements, as well as those who are self-employed. It is hoped that recently passed changes to Fair Work legislation in Australia will result in better standards for ‘employee-like workers’ in the gig economy (Grattan, 2023).

The diversity among the informal worker cohort in this study hints at the pervasiveness of informal employment in Australian communities, and their unconventional labour-force status is likely to mean that informal employment may be more common than reported. In Australia, the likely growth of informal employment is a matter of concern for health promoters who are charged with advocating for supportive environments in their pursuit of a socioecological approach to health. Informal employment is a particular type of ‘precarious employment’ in that it is not only insecure but also makes workers invisible, which exposes them to multiple health risks. Further, informal workers often experience social precarity in other areas of their lives, further compromising their health and well-being.

Informal employment offers an opportunity to access important economic resources, but employment needs to be more than a means through which material needs are met. Any perceived benefits of informal employment are tempered by indecent employment conditions of low wages, exploitation, the weight of invisibility, and lack of protection and voice.

This study adds to the sparse literature on informal work and health in high-income countries. Employment conditions are widely recognized as social and commercial determinants of health but most commonly many health promoters assume the main risks from work are those recognized through a traditional OHS lens. Informal work, however, poses those risks but in addition creates considerable psychosocial risks which health promoters in all countries need to take into account in devising healthy employment and social protection policies and practices.

## Data Availability

The data underlying this article cannot be shared publicly to protect the privacy of individuals that participated in the study. The data will be shared on reasonable request to the corresponding author.
